# Prevalence and Multidrug Resistance of WHO-Priority Bacterial Pathogens in a Romanian Intensive Care Unit

**DOI:** 10.3390/jcm15072799

**Published:** 2026-04-07

**Authors:** Alina Simona Bereanu, Bogdan Ioana Vintilă, Lilioara-Alexandra Oprinca-Muja, Rareș Bereanu, Ioana Roxana Codru, Raluca Maria Bădilă, Sandra Ioana Neamțu, Cosmin Ioan Mohor, Liiana Carmen Prodan, Mihai Sava

**Affiliations:** 1 Faculty of Medicine, Lucian Blaga University of Sibiu, Lucian Blaga Street 2A, 550169 Sibiu, Romania; alina.bereanu@ulbsibiu.ro (A.S.B.); rares.bereanu@ulbsibiu.ro (R.B.); ioanaroxana.bera@ulbsibiu.ro (I.R.C.); cosmin.mohor@ulbsibiu.ro (C.I.M.); liianacarmen.prodan@ulbsibiu.ro (L.C.P.); mihai.sava@ulbsibiu.ro (M.S.); 2 County Clinical Emergency Hospital, Bld. Corneliu Coposu, nr. 2-4, 550245 Sibiu, Romania; ralucamaria.vingerzan@ulbsibiu.ro (R.M.B.); sandraioana.neamtu@ulbsibiu.ro (S.I.N.)

**Keywords:** WHO 2024 BPPL, ESKAPEE, MDR bacteria, biofilms, carbapenem-resistant *Klebsiella pneumoniae*, Romania prevalence, intensive care infections, MRSA

## Abstract

**Background/Objectives**: The rise in healthcare-associated infections caused by multidrug-resistant (MDR) bacteria in hospitals, particularly in intensive care units, has resulted in increased rates of morbidity and mortality, escalating costs, and has become a significant public health concern. In our Intensive Care Unit, we address healthcare-associated infections caused by multidrug-resistant bacteria, with a specific focus on those listed in the WHO 2024 List of Critically and Highly Prioritized Pathogens. **Methods**: Over the course of 1 year, from 1 January to 31 December 2024, we monitored the prevalence of healthcare-associated infections in the Intensive Care Unit of the Sibiu County Emergency Clinical Hospital, Romania, and the antibiotic susceptibility of the isolated bacteria. **Results**: The majority of infections were caused by pathogens in the ESKAPEE group. The most frequently isolated microorganism was *Klebsiella pneumoniae* (36.8%), followed by *Acinetobacter baumannii* (24.5%), classified as a critical priority by the WHO in 2024. Most positive samples for critical priority pathogens, including *Klebsiella pneumoniae* and *Acinetobacter baumannii*, as well as all MRSA strains (high priority), were obtained from tracheal aspirates collected from intubated and mechanically ventilated patients. A significant proportion of the isolated bacteria were multidrug-resistant, including extensively drug-resistant and pan-drug-resistant strains. **Conclusions**: The increase in antibiotic and antimicrobial resistance among hospital strains raises serious concerns about limited treatment options.

## 1. Introduction

The rise in healthcare-associated infections caused by multidrug-resistant (MDR) bacteria in hospitals, particularly in intensive care units, has resulted in limited treatment options, increased rates of morbidity and mortality, escalating costs, and has become a significant public health concern. Statistics show that in 2019, antibiotic resistance was directly responsible for 1.27 million deaths, with an additional 4.95 million deaths associated with it. By 2021, antimicrobial resistance was linked to 4.71 million deaths, including 1.14 million deaths directly caused by it [1,2,3].

Most hospital-acquired infections in intensive care units are from the ESKAPEE group (*Enterococcus faecium*, *Staphylococcus aureus*, *Klebsiella pneumoniae*, *Acinetobacter baumannii*, *Pseudomonas aeruginosa*, *Enterobacter*, *Escherichia coli*), known for their strong antibiotic resistance. Critical pathogens include carbapenem-resistant *A. baumannii*, *Enterobacterales*, and rifampicin-resistant Mycobacterium tuberculosis. High-priority pathogens are fluoroquinolone-resistant Salmonella Typhi, vancomycin-resistant *E. faecium*, and methicillin-resistant *S. aureus*. Medium-priority pathogens include macrolide-resistant group A and B Streptococcus, while third-generation cephalosporin-resistant *Enterobacterales* is now a distinct critical pathogen, and carbapenem-resistant *P. aeruginosa* has been downgraded to high-priority status [4,5,6,7,8,9,10].

Multidrug-resistant Gram-negative bacteria primarily develop resistance through the production of beta-lactamases, particularly carbapenemases [11,12,13,14]. KPC-producing *Enterobacteriaceae* are common in Italy, Greece, Israel, Argentina, Colombia, and the US. NDM-1 producers are mostly found in India, Sri Lanka, and Pakistan, while OXA-48-like producers are prevalent in Turkey, Malta, and parts of North Africa and the Middle East [15,16,17,18].

The 2016 European Antibiotic Surveillance Network report indicated an average carbapenem resistance of 6.1%, with higher values in Italy, Greece, and Romania [18]. In 2022, Greek authorities reported an alarming spread of the carbapenemase-producing *K. pneumoniae* ST39 strain [19]. According to a recent report by the European Antimicrobial Resistance Surveillance Network, the estimated total incidence of carbapenem-resistant *K. pneumoniae* infections increased by 61% from 2019 to 2024, and the incidence of infections caused by third-generation cephalosporin-resistant *E. coli* increased by 5.9% [20].

The spread of multidrug-resistant *K. pneumoniae* in intensive care units is concerning, as it is found in patient secretions and hospital biofilms. This pathogen causes ventilator-associated pneumonia and other healthcare-associated infections. Its resistance mechanisms include gene accumulation via horizontal transfer, enzymatic inactivation of antibiotics, target modification, increased efflux pump activity, and biofilm formation [10,21,22].

*A. baumannii* develops antibiotic resistance through genetic flexibility, which enables rapid mutation and the mobilization of genetic elements, particularly plasmids. It primarily combats antibiotics by reducing membrane permeability, activating efflux pumps, and modifying antibiotic targets. Additionally, its capacity to form biofilms on medical devices poses a serious risk, particularly in intensive care units [23,24].

*P. aeruginosa* exhibits strong intrinsic antibiotic resistance, employing efflux pumps to expel antibiotics and possessing a low-permeability outer membrane. It can acquire resistance via gene transfer and mutations and readily colonizes medical devices and hospital surfaces, forming persistent biofilms [25,26].

Gram-positive bacteria employ two main strategies to develop resistance: producing beta-lactamases that degrade antibiotics or modifying the target, namely penicillin-binding proteins (PBPs), either through acquisition of exogenous DNA or through genetic mutations, thereby decreasing antibiotic affinity and increasing treatment resistance. Among the most important multidrug-resistant Gram-positive bacteria are methicillin-resistant *S. aureus* (MRSA) and vancomycin-resistant *E. faecium* (VRE) [27,28,29].

Antimicrobial resistance is a significant global public health issue. The WHO’s Global Antimicrobial Resistance and Use Surveillance System (GLASS) monitors this issue. In its December 2022 report, based on 2020 data from 87 countries, it noted rising antimicrobial resistance levels since 2017, particularly high carbapenem resistance in *K. pneumoniae* and *Acinetobacter* spp. in blood infections, raising concerns about treatment failure [30,31].

The main objectives of international guidelines include preventing patient colonization, avoiding progression from colonization to infection, and limiting the transmission of MDR bacteria [32,33,34,35,36,37]. The high resistance is due to both the limited diffusion of antimicrobial substances within the biofilm and the phenotypic and genotypic characteristics of the bacteria living within it, which differ from those of planktonic forms [38,39,40,41].

Bacterial biofilm formation involves several stages. First, bacteria attach to a surface and stabilize their attachment by secreting adhesive substances, forming microcolonies. As they mature, bacteria communicate through quorum sensing, increasing biofilm complexity. Eventually, the mature biofilm releases bacteria, allowing them to disperse and colonize new areas, contributing to infection spread [39,40,41,42,43,44,45,46,47,48,49,50,51,52,53]. Between 60% and 80% of microbial infections are associated with the formation of bacterial biofilms [51,54,55]. The primary bacteria in biofilm-related infections on medical devices include *Staphylococcus* species and multidrug-resistant Gram-negative bacteria such as *K. pneumoniae*, *P. aeruginosa*, *A. baumannii*, and *E. coli*. In intensive care units, ventilator-associated pneumonia commonly involves Gram-negative aerobic bacteria—especially *K. pneumoniae*, *Acinetobacter species*, and *P. aeruginosa*—found in over 60% of cases, alongside Gram-positive cocci like MRSA [7,43,46,56,57,58,59,60,61,62]. Risk factors for colonization and infection with MDR bacteria include inappropriate antibiotic therapy, prolonged hospitalization in intensive care units, and comorbidities [17,53,63,64,65].

Another category of healthcare-associated infections in intensive care units is infections associated with intravascular catheters. The most commonly implicated bacteria are *S. aureus*, *E. faecalis*, *P. aeruginosa*, and *K. pneumoniae* [43,54,63]. A 2020 trans-European surveillance report reported a rate of 4.9 bloodstream infections associated with central venous catheters per 1000 catheter days [66]. In the last decade, several practices related to central venous catheterization have changed, reducing infectious complications; some studies suggest potential benefits of scheduled CVC replacement, particularly for non-subclavian catheters [67,68,69,70,71,72,73,74,75,76,77].

Healthcare-associated infections with multidrug-resistant (MDR) bacteria are common in intensive care units due to the fragility of critically ill patients, comorbidities, immunosuppression, and the extensive use of invasive devices and prolonged antibiotic treatment. Despite advancements in technology and new antibiotics, mortality remains high. The death rate among patients with bacteremia or bronchopneumonia caused by carbapenemase-producing *K. pneumoniae* ranges from 30% to 70% [18,19,78,79,80].

Our paper aims to emphasize the threat posed by antimicrobial resistance and to improve understanding of resistance mechanisms and transmission routes. Such knowledge can better inform infection-control strategies, supporting the development and implementation of local protocols aligned with international guidelines. In our Intensive Care Unit, this challenge is particularly significant: multidrug-resistant bacterial infections are frequently encountered, with carbapenemase-producing *K. pneumoniae*, a WHO-designated critical-priority pathogen, among the most commonly isolated organisms from samples of critically ill patients.

## 2. Materials and Methods

Clinical specimens included tracheal aspirates, bronchial aspirates, central venous catheter tips, blood cultures, pharyngeal exudates, wound secretions (from surgical wounds, abscesses, and superinfected venous ulcers), urine cultures, and pleural fluid. The classification of cases as infections and their reporting as healthcare-associated infections were conducted by the attending physician in collaboration with the Infection Prevention and Control Service of the Sibiu County Emergency Clinical Hospital. The reporting cases of infection were made in accordance with the case definitions outlined in European Commission Decision 945/2018, dated 22 June 2018, which addresses communicable diseases and related public health issues requiring epidemiological surveillance; these case definitions are also regulated by law in our country [81]. Descriptive statistics were used to summarize categorical variables as frequencies and percentages. Due to the descriptive nature of the study, no inferential statistical tests were applied.

### 2.1. Sample Collection and Bacterial Isolation

The present study was conducted over 1 year, from January 2024 to December 2024, involving patients admitted to the Intensive Care Unit of the Sibiu County Emergency Clinical Hospital. This study protocol was approved by the Ethics Committee of the Sibiu County Clinical Emergency Hospital (Approval No. 26995/13 November 2023). Isolates were identified using traditional biochemical methods, including Triple Sugar Iron (TSI) agar, Motility Indole Urease (MIU) tests, and Simmons citrate agar. Additional identification techniques included Gram and methylene blue staining, as well as the automated Vitek 2 Compact identification system. In some instances, molecular biology tests were conducted on samples collected from the upper respiratory tract. The tests used the GeneXpert system, which detects COVID-19, influenza, and respiratory syncytial virus, and the BioFire panel, which identifies pathogens responsible for upper and lower respiratory infections, sepsis, meningitis, joint infections, gastrointestinal infections, and tropical fever.

### 2.2. Antimicrobial Susceptibility Testing

Currently, our laboratory adheres to the standards established by the Clinical and Laboratory Standards Institute (CLSI—2024, M100, 34th edition). There were two main methods for evaluating antibacterial activity: the diffusion method (Kirby–Bauer) and the dilution method, which allows for the precise determination of the Minimum Inhibitory Concentration (MIC).

Kirby–Bauer method. This method involves seeding the test bacteria onto the surface of Mueller–Hinton solid medium, followed by the application of antibiotic micro-tablets to the medium surface, in accordance with CLSI standards. The antibiotic diffuses into the culture medium, gradually decreasing in concentration as it moves away from the disc. The bacteria grow uniformly across the medium until they reach the area where the antibiotic concentration equals the MIC. The diameters of the inhibition zones are measured and compared with international reference values (CLSI) to determine the sensitivity or resistance category.

Automated antibiogram—Vitek 2 Compact. This system allows both the identification of bacteria and fungi and the testing of their antibiotic sensitivity using special cards (GP card—Gram-positive bacteria, GN card—Gram-negative bacteria, YST card—yeast, ANC—anaerobic bacteria and *Corynebacteria*, NH—*Neisseria* and *Haemophilus*). The reading is performed using the turbidimetric method. The system includes the Advanced Expert System (AES) software (MYLA software, version 4.9, Biomerieux, l’Étoile, France), which validates results by referencing a complex database.

Antibiogram by broth microdilution. This is the standardized method for assessing antibiotic sensitivity and is used in our laboratory only for cases of MDR, XDR, and PDR infections. During the period analyzed, the method was applied exclusively for colistin testing.

Resistance classifications follow the international definitions proposed by Magiorakos et al. [67]: MDR—multidrug-resistant, XDR—extensively drug-resistant, and PDR—pan-drug-resistant.

## 3. Results

**Figure 1:**
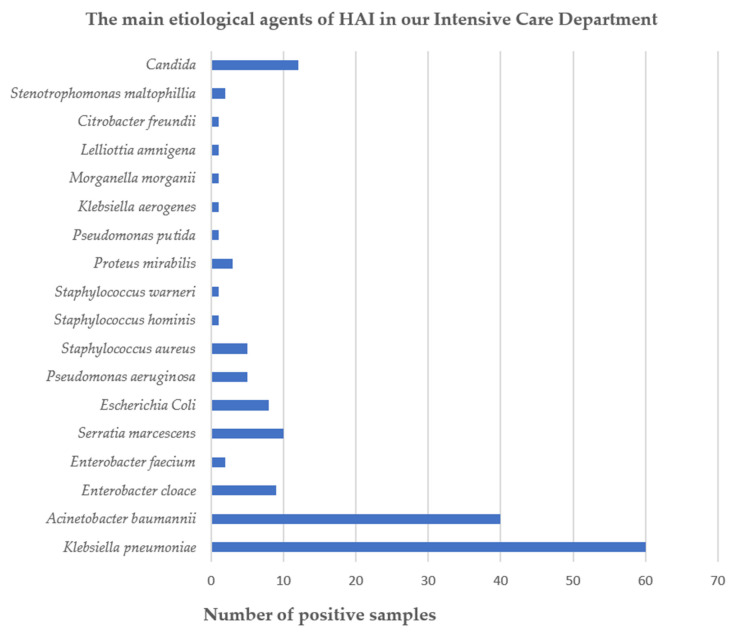
The main etiological agents of healthcare-associated infections in our intensive care unit. HAI—Healthcare-associated infections.

Over the course of one year, from 1 January to 31 December 2024, we monitored the prevalence of healthcare-associated infections in the Intensive Care Unit of the Sibiu County Emergency Clinical Hospital and the antibiotic susceptibility of the isolated bacteria. The majority of infections were caused by pathogens in the ESKAPEE group. The most frequently isolated microorganism was *K. pneumoniae* (36.8%), followed by *A. baumannii* (24.53%). Other Gram-negative bacteria included *E. cloacae* (5.53%), *E. coli* (4.9%), and *P. aeruginosa* (3%). Gram-positive cocci were identified less often, with *S. aureus* accounting for 3% and *E. faecium* for 1.22%. In total, 163 microorganisms were identified during the study period, of which 151 (92.6%) were bacterial species and 12 (7.4%) were fungal species (Figure 1, Table 1 and Table 2). Overall, the pathogens with the highest incidence were *K. pneumoniae*—a WHO 2024 BPPL critical-priority agent—representing 36.8% of isolates, followed by *A. baumannii*, also classified as a critical-priority pathogen, at 24.53%. A total of 163 hospital-acquired infections were identified according to the definitions outlined in the Materials and Methods section. These infections occurred in 88 patients, with some patients experiencing more than one HAI, either as coinfections at the same anatomical site or as infections involving different anatomical sites.

**Figure 2:**
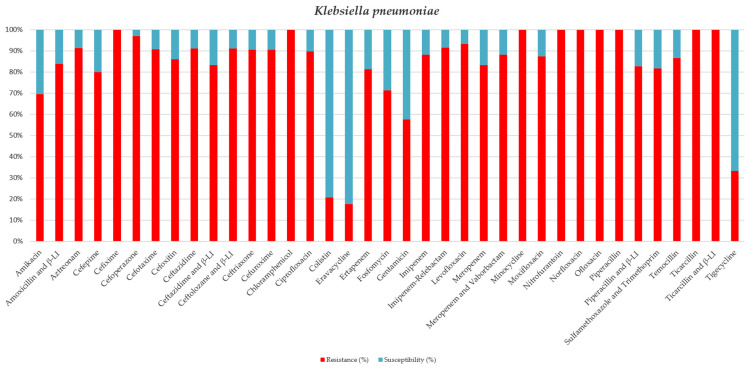
Antibiotic sensitivity results for *K. pneumoniae* strains isolated from critically ill patients between 1 January 2024 and 31 December 2024, in the Intensive Care Unit of the County Clinical Emergency Hospital of Sibiu; red indicates the percentage of resistant strains, while blue indicates the percentage of sensitive strains; β-LI = beta-lactamase inhibitor.

We present the incidence of bacteria classified by the WHO as critical and high-priority, isolated from patients’ secretions admitted to our Intensive Care Unit, along with their antibiotic resistance patterns for 2024 (see Tables and Figures). Most of the isolated bacteria (68.09%) associated with hospital-acquired infections were resistant to antibiotics, including ESBL, MDR, XDR, and even PDR strains. Among the *K. pneumoniae* strains isolated, 37 (61.66%) were classified as XDR, while 5 (8.33%) were classified as PDR (refer to Table 3 and Figure 2). Notably, over 80% of these strains were resistant to carbapenems.

**Figure 3:**
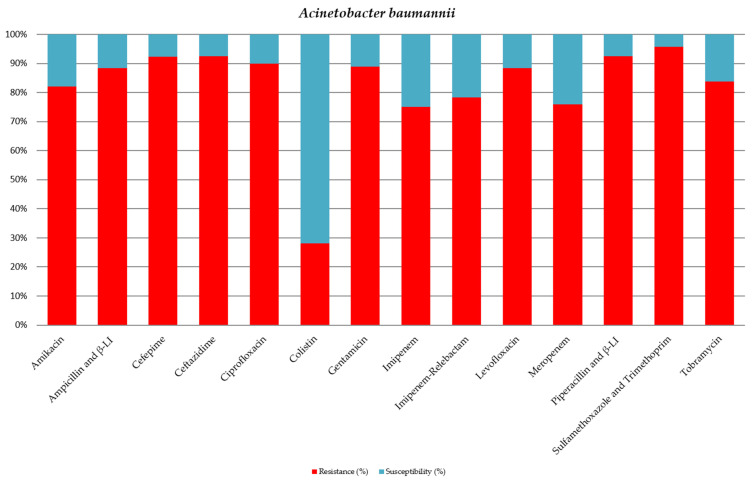
Antibiotic sensitivity results for *A. baumannii* strains isolated from critically ill patients between 1 January 2024 and 31 December 2024, in the Intensive Care Unit of the County Clinical Emergency Hospital of Sibiu; red indicates the percentage of resistant strains, while blue indicates the percentage of sensitive strains; β-LI = beta-lactamase inhibitor.

Of the isolated strains of *A. baumannii*, 28 strains (70%) were classified as XDR, while five strains (12.5%) were classified as PDR (Table 4, Figure 3). The majority of strains (over 70%) exhibited carbapenem resistance.

**Figure 4:**
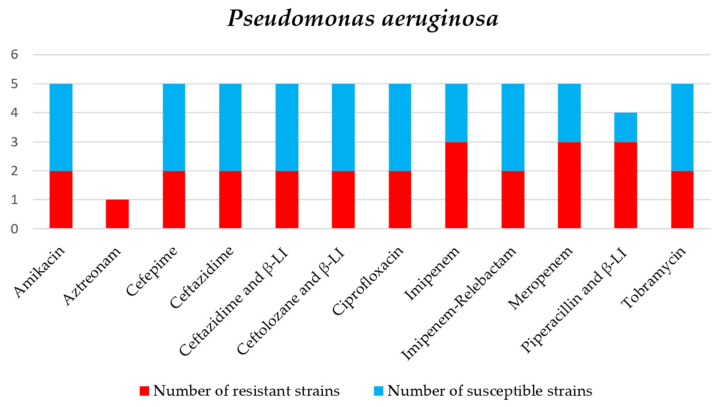
Antibiotic sensitivity results for *P. aeruginosa* strains isolated from critically ill patients between 1 January 2024 and 31 December 2024, in the Intensive Care Unit of the County Clinical Emergency Hospital of Sibiu; red indicates the number of resistant strains, while blue indicates the number of sensitive strains; β-LI = beta-lactamase inhibitor.

Regarding *P. aeruginosa*, 2 of 5 isolates (40%) were classified as XDR, both obtained from tracheal aspirates (see Table 5 and Figure 4). The rate of carbapenem resistance was about 60% among these strains, which is lower than that observed in *K. pneumoniae* and *A. baumannii*.

**Figure 5:**
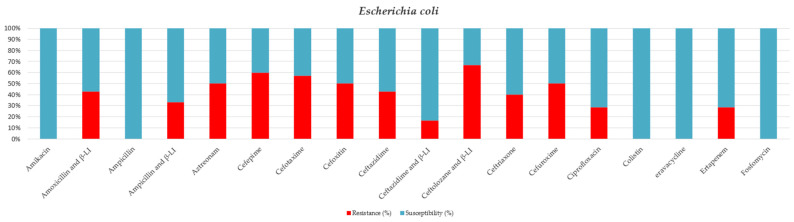
Antibiotic sensitivity results for *E. Coli* strains isolated from critically ill patients between 1 January 2024 and 31 December 2024, in the Intensive Care Unit of the County Clinical Emergency Hospital of Sibiu; red indicates the percentage of resistant strains, while blue indicates the percentage of sensitive strains; β-LI = beta-lactamase inhibitor.

Most of the isolated *E. coli* strains did not show any pattern of antibiotic resistance (75%) (Table 6 and Figure 5).

**Figure 6:**
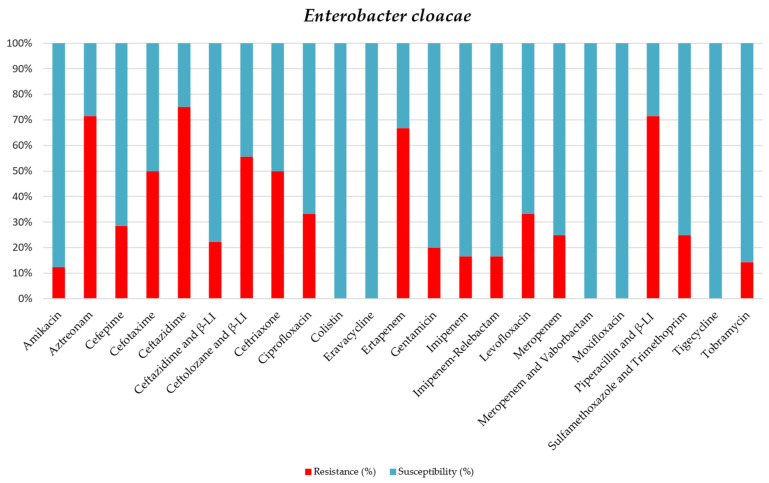
Antibiotic sensitivity results for E. cloacae strains isolated from critically ill patients between 1 January 2024 and 31 December 2024, in the Intensive Care Unit of the County Clinical Emergency Hospital of Sibiu; red indicates the percentage of resistant strains, while blue indicates the percentage of sensitive strains; β-LI = beta-lactamase inhibitor.

Out of the nine strains of *E. cloacae*, one strain was classified as XDR (11.1%), and another was classified as PDR (11.1%) (Table 7 and Figure 6).

**Figure 7:**
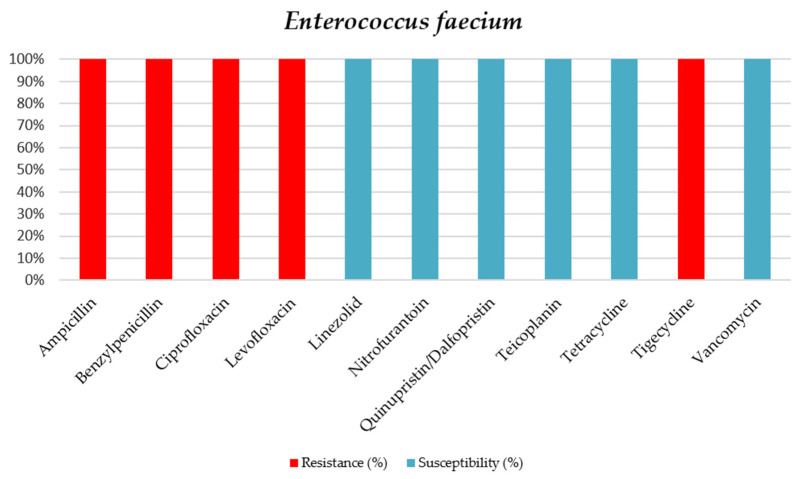
Antibiotic sensitivity results for *E. faecium* strains isolated from critically ill patients between 1 January 2024 and 31 December 2024, in the Intensive Care Unit of the County Clinical Emergency Hospital of Sibiu; red indicates the percentage of resistant strains, while blue indicates the percentage of sensitive strains.

Only two *E. faecium* samples were positive, and both showed no resistance to Vancomycin (see Table 8 and Figure 7).

Of the five samples positive for *S. aureus* (all tracheal aspirates), four samples were positive for MRSA (80%) (Table 9).

**Figure 8:**
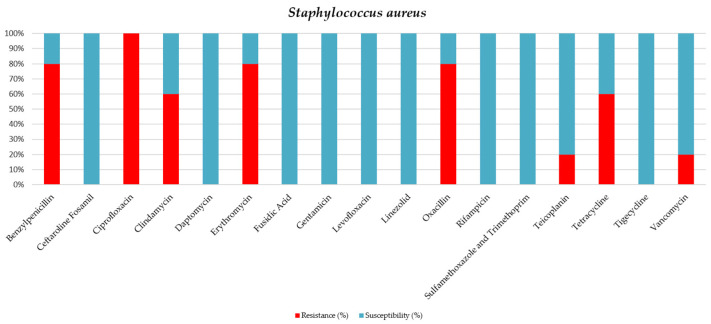
Antibiotic sensitivity results for *S. aureus* strains isolated from critically ill patients between 1 January 2024 and 31 December 2024, in the Intensive Care Unit of the County Clinical Emergency Hospital of Sibiu; red indicates the percentage of resistant strains, while blue indicates the percentage of sensitive strains.

Out of a total of 163 positive samples collected from patients, 95 (58.28%) were tracheal and bronchial aspirate samples. The majority of these positive tracheal and bronchial aspirates were *K. pneumoniae* (32 samples) and *A. baumannii* (30 samples) (see Table 2 and Table 3). All samples that tested positive for *S. aureus* were from tracheal aspirates. Methicillin-resistant *S. aureus* accounted for 80% of *S. aureus* cases, as 4 of 5 tracheal aspirate samples were positive for S. aureus and were MRSA (see Table 8 and Figure 8).

Six positive samples were identified from the tips of central venous catheters extracted from patients and sent to the Microbiology Laboratory for analysis. Among these six samples, two tested positive for *A. baumannii*, two for *K. pneumoniae*, and two for *E. cloacae* (see Table 2, Table 3 and Table 6). No central venous catheter-related infections were identified in these patients, as peripheral blood cultures were negative. These findings were classified as colonizations of the central venous catheters. Additional positive results included 21 urine cultures and 19 blood cultures. Furthermore, 18 samples collected from surgical wounds, abscesses, and superinfected varicose ulcers tested positive for various microorganisms (see Table 10).

A significant proportion (68.09%) of the bacteria isolated from samples collected from critically ill patients exhibited antibiotic resistance, including MDR, XDR, and even PDR. Among the five samples positive for *S. aureus* (all tracheal aspirates), four were identified as MRSA, representing 80% of the samples.

Among antibiotic-resistant bacterial isolates, a very high percentage of XDR (60.36%) and PDR (9.9%) strains were observed (Table 11). The bacteria were resistant to all antibiotics tested (PDR), including five strains of *K. pneumoniae*, five strains of *A. baumannii*, and one strain of *E. cloacae.*

## 4. Discussion

In our Intensive Care Unit, we address healthcare-associated infections caused by multidrug-resistant bacteria, with a specific focus on those listed in the WHO 2024 List of Critically and Highly Prioritized Pathogens [8,9]. Among the microorganisms isolated in our unit, *K. pneumoniae* was the most frequently identified. Of all isolated organisms (bacteria and fungi), 36.8% were *K. pneumoniae*, primarily isolated from tracheal aspirates of patients. A smaller percentage came from other sources, such as blood cultures, urine cultures, wound secretions, and invasive medical devices (central venous catheters), used for critically ill patients. The second most commonly isolated organism in our department is *A. baumannii*, which, like *K. pneumoniae*, was primarily isolated from tracheal aspirates of these patients.

According to the World Health Organization’s 2024 Priority Pathogen List, antibiotic-resistant Gram-negative bacteria, including *K. pneumoniae*, *Acinetobacter* spp., and *E. coli*, were assigned to the highest-priority quartile. Notably, carbapenem-resistant *K. pneumoniae* ranked first, receiving a priority score of 84%. In our study, we found that over 80% of *K. pneumoniae* strains and more than 70% of *A. baumannii* strains exhibited carbapenem resistance. *P. aeruginosa* demonstrated a 60% resistance rate to carbapenems. In fact, in 2024, the WHO reclassified *P. aeruginosa* from a critically prioritized pathogen to a high-priority pathogen, indicating a decrease in resistance compared to its 2017 classification [8,9].

Among Gram-positive bacteria, the highest rankings in the WHO’s BPPL 2024 are Vancomycin-resistant *E. faecium* (69%) and Methicillin-resistant *S. aureus* (59%). In our unit, we isolated two *Enterococcus* strains from patients; neither showed resistance to Vancomycin. Regarding *S. aureus* isolates from our patients, although they accounted for a low incidence relative to the total number of microorganisms isolated, the majority (80%) were identified as MRSA. All MRSA strains were sourced from tracheal aspirates [8,9].

Most positive *K. pneumoniae* samples were collected from tracheal aspirates (48.33%). Similarly, 67.5% of positive *A. baumannii* samples were from tracheal aspirates. The risk of ventilator-associated pneumonia increases exponentially in patients who have been mechanically ventilated for more than 48 h, making it the most common healthcare-associated infection in intensive care units. The endotracheal tube is believed to be the primary source of VAP, as it disrupts the upper respiratory tract’s natural defense mechanisms, allowing pathogens to invade the tracheobronchial tree. One mechanism of bacterial resistance to antibiotics is biofilm formation on biotic and abiotic surfaces, including medical devices and hospital surfaces. Many microorganisms can easily adhere to the endotracheal tube, forming biofilms and colonizing both the tube and the patient’s mucosa. The transition from colonization to infection poses a significant threat, particularly because biofilm-associated microorganisms are highly resistant to antibiotics and other antimicrobials. Despite technological advancements, identifying multi-microbial biofilms remains challenging. It is also difficult to distinguish between an actual infection and colonization of the patient’s mucosa or intubation tube. This complicates the etiological diagnosis of VAP and may hinder the implementation of an appropriate therapeutic regimen [46,59,60,61,68].

The primary cause of VAP is typically bacterial, with Gram-negative bacteria such as *K. pneumoniae*, *Acinetobacter species*, *P. aeruginosa*, *Enterobacter* spp., *Stenotrophomonas maltophilia*, and *Serratia marcescens* accounting for the majority of cases (approximately 60%). Additionally, Gram-positive cocci, particularly methicillin-resistant *S. aureus*, are also significant contributors [46,58,60,61]. In our department, VAP was the most common healthcare-associated complication, and its etiology was consistent with findings reported in the literature. Of the 163 positive samples collected from patients, 98 (60.12%) were obtained from tracheal and bronchial aspirates. Biofilms serve as reservoirs for multidrug-resistant pathogens. The internal organization of these biofilms, along with processes such as the horizontal transfer of resistance genes and genetic mutations, contributes to an increased resistance to disinfectants, antimicrobials, and antibiotics. Conversely, the widespread use of antimicrobials (including antibiotics) or repeated inadequate disinfection can further promote bacterial resistance [69,70,71,72,73,74,75,76,77,80,82,83,84,85,86,87,88,89]. MDR bacteria easily form biofilms on invasive medical devices such as catheters, drainage tubes, and endotracheal tubes. They can also develop on the hands of medical staff and on surfaces in the hospital environment, known as dry-surface biofilms. Multidrug-resistant Gram-negative bacteria, including *K. pneumoniae*, *A. baumannii*, *P. aeruginosa*, and *E. coli*, are frequently found in biofilms on urinary catheters and central venous catheters [41,45,46,90,91,92,93,94,95,96,97]. In 2021, Folliero and colleagues published a study that focused on identifying the primary microorganisms that colonize medical devices, their capacity to form biofilms, and the prevalence of multidrug-resistant strains within those biofilms. They found that 72.7% of *K. pneumoniae* strains produced biofilms. This bacterium was commonly found in urinary catheters, nephrostomy tubes, abdominal drains, and central venous catheters. Moreover, some biofilms from medical devices contained more than one type of microorganism. Regarding antibiotic resistance, 59.2% of the isolated strains were classified as multidrug-resistant [41].

In our unit, six positive samples were identified from central venous catheter tips extracted from patients and sent to the microbiology laboratory for analysis. These samples account for 3.68% of the total number of positive cultures isolated. Among these, two samples tested positive for *A. baumannii*, two for *K. pneumoniae*, and two for *E. cloacae*, which were found to be sensitive to Vancomycin. Furthermore, no central venous catheter-related bloodstream infections (CRBSI) were detected in these patients, as blood cultures obtained simultaneously from a peripheral vein were negative.

Over the past decade, practices related to central venous catheter placement have evolved, leading to a reduction in both infectious and non-infectious complications. Using a 2% chlorhexidine alcohol solution for skin preparation before catheter placement has been shown to significantly reduce the risk of catheter-related infections compared with a 5% povidone-iodine solution. Guidelines now recommend ultrasound-guided techniques for CVC insertion, and these methods have become standard in many medical centers, including our unit. Ultrasound-guided CVC placement allows for access to the internal jugular vein at its more proximal region or to the brachiocephalic vein. This approach facilitates a better seal of the chlorhexidine dressing, thereby reducing the risk of infection from dressing discontinuity. Numerous studies have demonstrated that transparent chlorhexidine-impregnated dressings are superior to standard dressings in reducing the risk of infection. Their use is recommended in international guidelines and is also standard practice in our Intensive Care Unit. Collectively, these advancements have significantly reduced the risk of infections associated with central venous catheters [66,98,99,100,101,102,103,104,105,106,107,108,109,110]. The REAREZO study group published the results in 2024, showing decreases in the incidence of CRBSI (fewer than 1 per 1000 CVC days) and CVC colonization (fewer than 7 per 1000 CVC days) [75].

The main objectives include preventing patient colonization and avoiding progression from colonization to invasive infection, because the mortality rate among patients with bacteremia or respiratory infections caused by MDR bacteria, especially carbapenemase-producing *K. pneumoniae*, remains high [97,98,99]. The materials used to manufacture invasive medical devices can impact bacterial adhesion and biofilm formation. This influence depends on several factors, including the hydrophobicity of the device material and the hydrophobicity of the bacterial cell wall. Thorarinsdottir and colleagues (2020) demonstrated that noble-metal-coated PVC endotracheal tubes significantly reduced the rate of bacterial biofilm formation compared to standard PVC tubes [84]. Polyvinyl chloride is hydrophobic, which helps explain why hydrophobic bacteria have a strong affinity for PVC endotracheal tubes, whereas noble metal-coated PVC tubes are hydrophilic. The cell wall of *K. pneumoniae* is also hydrophobic, further contributing to its tendency to adhere to PVC tubes [91,92,93,97]. Most endotracheal tubes used in our intensive care unit are made of PVC, which accounts for the high rate of tracheal aspirates that test positive for *K. pneumoniae*.

Since 2011, the CDC has highlighted that polyurethane CVCs have a lower infection rate than those made of polyvinyl chloride or polyethylene. The guidelines also recommend using CVCs impregnated with chlorhexidine/silver sulfadiazine or minocycline/rifampin—impregnated CVC in patients whose catheter is expected to remain in place more than five days. This recommendation remains in place today [98].

Dry-surface biofilms are found in hospital settings, particularly in Intensive Care Units near patients, such as on mattresses and bed rails. However, they can also be found farther away on frequently touched surfaces like door handles, light switches, trolley handles, ceilings, curtains, keyboards, and window sills. DSBs are resistant to common disinfectants and antimicrobials, which contributes to the spread of healthcare-associated infections. Additionally, polymicrobial biofilms are more resistant to disinfection than monomicrobial biofilms [111,112,113,114,115,116]. Dry biofilms on frequently touched surfaces in Intensive Care are a significant reservoir for pathogens [92,117]. In 2023, Centeleghe and colleagues published a study confirming that *K. pneumoniae* can survive on dry surfaces as dried bacterial cells for an extended period. The study found that this bacterium can remain viable on a dry surface for up to 4 weeks, even as its culturability declines [118].

*K. pneumoniae* MDR, classified by the WHO in 2024 as a critical priority with the highest priority score, is frequently encountered in our Intensive Care Unit. This pathogen has special abilities that enable it to survive extreme physical conditions, readily form biofilms, and rapidly acquire resistance to antibiotics and other antimicrobials.

This study has several limitations that should be acknowledged. First, the retrospective and single-center design limits the generalizability of the findings and restricts causal interpretations. Consequently, a comprehensive characterization of the underlying resistance mechanisms was not possible in our setting, limiting the ability to correlate phenotypic resistance profiles with specific genetic determinants. Additionally, molecular epidemiological analyses, such as multilocus sequence typing or whole-genome sequencing, were not performed; therefore, it was not possible to determine whether the high resistance rates observed were due to clonal dissemination or to independent emergence under antibiotic selective pressure. Finally, the study primarily provides a descriptive analysis of ICU pathogens, and the absence of longitudinal clinical correlations or systematic characterization of resistance genes limits the ability to assess the temporal dynamics of antimicrobial resistance or the impact of specific clinical interventions.

## 5. Conclusions

This study describes the microbiological profile and antimicrobial resistance patterns of healthcare-associated infections in the Intensive Care Unit of the Sibiu County Clinical Emergency Hospital during 2024. A total of 163 isolates were identified, predominantly Gram-negative bacteria from the ESKAPEE group. *Klebsiella pneumoniae* (36.8%) and *Acinetobacter baumannii* (24.5%) were the most frequently isolated pathogens, most commonly recovered from tracheal and bronchial aspirates associated with ventilator-associated pneumonia. A high proportion of bacterial isolates (68.1%) exhibited antimicrobial resistance, including ESBL, MDR, XDR, and PDR phenotypes. XDR strains represented the largest category, particularly among *K. pneumoniae* and *A. baumannii*, which also showed high rates of carbapenem resistance. MRSA was identified in 80% of *Staphylococcus aureus* isolates. These findings highlight the predominance of multidrug-resistant Gram-negative pathogens among healthcare-associated infections in our ICU and provide a local antimicrobial resistance profile that may support infection surveillance and antimicrobial stewardship strategies.

## Figures and Tables

**Table 1:** Overall distribution of microorganisms isolated in the ICU (2024).

Microorganism	Number of Isolates (n)	Percentage (%)	WHO 2024 BPPL Priority
*Klebsiella pneumoniae*	60	36.8	Critical
*Acinetobacter baumannii*	40	24.5	Critical
*Enterobacter cloacae*	9	5.5	Critical (3GC-resistant *Enterobacterales*)
*Escherichia coli*	8	4.9	Critical (3GC-resistant *Enterobacterales*)
*Pseudomonas aeruginosa*	5	3.0	High
*Staphylococcus aureus*	5	3.0	High (MRSA)
*Enterococcus faecium*	2	1.2	High (VRE)
Fungal species	12	7.4	Not classified
Total	163	100	—

**Table 2:** Distribution of antimicrobial resistance profiles among bacterial isolates.

Resistance Profile	Number of Isolates (n)	Percentage (%)
No resistance detected	48	31.8
ESBL	14	9.3
MDR	19	12.6
XDR	67	44.4
PDR	11	7.3
Total bacterial isolates	151	100

**Table 3:** *K. pneumoniae* isolated from critically ill patients admitted to the Intensive Care Unit of the County Clinical Emergency Hospital, Sibiu, Romania, between 1 January 2024 and 31 December 2024.

*K. pneumoniae* Sample Origin and Resistance Pattern	Number of Isolates
**Bronchial aspirate**	**3**
No resistance pattern	2
XDR-positive	1
**Tracheal aspirate**	**29**
No resistance pattern	2
ESBL-positive	4
MDR-positive	2
PDR-positive	1
XDR-positive	20
**Catheter tip**	**2**
ESBL-positive	2
**Pharyngeal swab**	**1**
ESBL-positive	1
**Surgical wound, abscess, ulcer, etc.**	**6**
ESBL-positive	2
MDR-positive	2
XDR-positive	2
**Blood sample**	**8**
MDR-positive	1
PDR-positive	2
XDR-positive	5
**Sputum**	**1**
XDR-positive	1
**Urine**	**10**
PDR-positive	2
XDR-positive	8
**Total**	**60**

**Table 4:** *A. baumannii* isolated from critically ill patients admitted to the Intensive Care Unit of the County Clinical Emergency Hospital, Sibiu, Romania, between 1 January 2024 and 31 December 2024.

*A. baumannii* Sample Origin and Resistance Pattern	Number of Isolates
**Bronchial aspirate**	**3**
XDR-positive	3
**Tracheal aspirate**	**27**
No resistance pattern	3
MDR-positive	2
PDR-positive	3
XDR-positive	19
**Catheter tip**	**2**
XDR-positive	2
**Surgical wound, abscess, ulcer, etc.**	**4**
MDR-positive	1
PDR-positive	1
XDR-positive	2
**Blood sample**	**3**
No resistance pattern	1
PDR-positive	1
XDR-positive	1
**Urine**	**1**
XDR-positive	1
**Total**	**40**

**Table 5:** *P. aeruginosa* isolated from critically ill patients admitted to the Intensive Care Unit of the County Clinical Emergency Hospital, Sibiu, Romania, between 1 January 2024 and 31 December 2024.

*P. aeruginosa* Sample Origin and Resistance Pattern	Number of Isolates
**Tracheal aspirate**	**3**
No resistance pattern	1
XDR-positive	2
**Surgical wound, abscess, ulcer, etc.**	**1**
No resistance pattern	1
**Blood sample**	**1**
No resistance pattern	1
**Total**	**5**

**Table 6:** *E. coli* isolated from critically ill patients admitted to the Intensive Care Unit of the County Clinical Emergency Hospital, Sibiu, Romania, between 1 January 2024 and 31 December 2024.

*E. coli* Sample Origin and Resistance Pattern	Number of Isolates
**Bronchial aspirate**	1
MDR-positive	1
**Tracheal aspirate**	3
No resistance pattern	3
**Surgical wound, abscess, ulcer, etc.**	1
No resistance pattern	1
**Urine**	3
No resistance pattern	2
ESBL-positive	1
**Total**	**8**

**Table 7:** *E. cloacae* isolated from critically ill patients admitted to the Intensive Care Unit of the County Clinical Emergency Hospital, Sibiu, Romania, between 1 January 2024 and 31 December 2024.

*E. cloacae* Sample Origin and Resistance Pattern	Number of Isolates
**Bronchial aspirate**	2
No resistance pattern	2
**Tracheal aspirate**	1
PDR-positive	1
**Catheter tip**	2
No resistance pattern	1
ESBL-positive	1
**Surgical wound, abscess, ulcer, etc.**	2
No resistance pattern	1
XDR-positive	1
**Urine**	2
No resistance pattern	1
ESBL-positive	1
**Total**	**9**

**Table 8:** *E. faecium* isolated from critically ill patients admitted to the Intensive Care Unit of the County Clinical Emergency Hospital, Sibiu, Romania, between 1 January 2024 and 31 December 2024.

*E. faecium* Sample Origin and Resistance Pattern	Number of Isolates
**Surgical wound, abscess, ulcer, etc.**	1
VRE-negative	1
**Urine**	1
VRE-negative	1
**Total**	2

**Table 9:** *S. aureus* isolated from critically ill patients admitted to the Intensive Care Unit of the County Clinical Emergency Hospital, Sibiu, Romania, between 1 January 2024 and 31 December 2024.

*S. aureus* Sample Origin and Resistance Pattern	Number of Isolates
**Tracheal aspirate**	5
No resistance pattern	1
MRSA-positive	4
Total	5

**Table 10:** Total number of positive samples isolated from critically ill patients admitted to the Intensive Care Unit of the County Clinical Emergency Hospital, Sibiu, Romania, between 1 January 2024 and 31 December 2024.

Sample Type	Type of Infection	Number of Samples (n)	Percentage (%)
Tracheal and bronchial aspirates	VAP	95	58.3
Blood cultures	HABSI	19	11.7
Urine cultures	UTI	21	12.9
Surgical wounds/abscesses/ulcers	SSI/SSTIs	18	11.0
Central venous catheter tips	CVC infection	6	3.7
Pharyngeal swab	HAP	2	1.2
Sputum	HAP	1	0.6
Pleural fluid	Empyema	1	0.6
Total		163	100

**Table 11:** Resistance profiles of positive samples isolated from critically ill patients admitted to the Intensive Care Unit of the County Clinical Emergency Hospital, Sibiu, Romania, between 1 January 2024 and 31 December 2024.

Resistance Profile	Number of Isolates
MDR-positive	19
XDR-positive	67
PDR-positive	11
ESBL-positive	14

## Data Availability

Data are contained within this article.
